# Global research hotspots and frontiers of myasthenia gravis from 2002 to 2021: A bibliometric study

**DOI:** 10.1097/MD.0000000000034002

**Published:** 2023-06-16

**Authors:** Jiali Yang, Jiaojiao Wu, Tingliang Han, Hua Lu, Fangcun Li, Leilei Li, Shaoting Su, Ping Jiang, Zhaomeng Hou

**Affiliations:** a Yancheng TCM Hospital Affiliated to Nanjing University of Chinese Medicine, Yancheng, China; b Xiangyu Pharmaceutical Co., Ltd., Linyi, China; c Guilin Municipal Hospital of Traditional Chinese Medicine, Guilin, China; d Guangxi University of Chinese Medicine, Nanning, China; e Shanghai University of Traditional Chinese Medicine, Shanghai, China.

**Keywords:** bibliometrics, CiteSpace, myasthenia gravis, visualization analysis, VOSviewer

## Abstract

The objective of this study is to utilize bibliometric and visual analysis techniques to identify hotspots and frontiers of research in myasthenia gravis (MG) and provide valuable references for future research. The Web of Science Core Collection (WoSCC) database was used to retrieve literature data related to MG research, which was then analyzed using VOSviewer 1.6.18, CiteSpace 6.1.R3, and the Online Platform for Bibliometric Analysis. The analysis revealed 6734 publications distributed across 1612 journals and contributed by as many as 24,024 authors affiliated with 4708 institutions across 107 countries/regions. The number of annual publications and citations for MG research has steadily increased over the past 2 decades, with the last 2 years alone witnessing a remarkable increase in annual publications and citations to over 600 and 17,000, respectively. In terms of productivity, the United States emerged as the top producing country, while the University of Oxford ranked first in terms of research institutions. Vincent A was identified as the top contributor in terms of publications and citations. *Muscle & Nerve* and *Neurology* ranked first in publications and citations respectively, with clinical neurology and neurosciences among the main subject categories explored. The study also identified pathogenesis, eculizumab, thymic epithelial cells, immune checkpoint inhibitors, thymectomy, MuSK antibodies, risk, diagnosis, and management as the current hot research topics in MG, while burst keywords like quality of life, immune-related adverse events (irAEs), rituximab, safety, nivolumab, cancer, and classification indicated the frontiers of MG research. This study effectively identifies the hotspots and frontiers of MG research, and offers valuable references for researchers interested in this area.

## 1. Introduction

Myasthenia gravis (MG) is an acquired autoimmune disease involving the neuromuscular junction (NMJ) mainly mediated by antibodies to acetylcholine receptors (AChR).^[1–4]^ In addition, antibodies against muscle specific tyrosine kinase (MuSK), low-density lipoprotein receptor-related protein 4 (LRP4), and ryanodine receptor may also be involved in MG pathogenesis by interfering with AChR aggregation, affecting AChR function and NMJ signaling.^[5–7]^ The typical clinical presentation of MG includes fluctuating muscle weakness and fatigue, worsened by activity and improved with rest, which can cause respiratory distress, myasthenic crisis, and, in severe cases, be life-threatening.^[8–11]^ Epidemiological studies have estimated the global prevalence of MG to be approximately 15 to 25 cases per 100,000 population, with an annual incidence rate of 8 to 10 cases per 1 million.^[12]^ The disease has a bimodal incidence pattern, affecting individuals of all ages but with a higher incidence among young women and elderly men.^[13–16]^ While the pathogenesis of MG remains unclear,^[17,18]^ available therapies for the condition include cholinesterase inhibitors, glucocorticoids, immunosuppressants, intravenous immunoglobulins, plasma exchange, and thymectomy.^[19–22]^ Emerging treatment options for MG include hematopoietic stem cell transplantation and targeted biologics, such as eculizumab and rituximab, which diversified the available treatment portfolio for MG patients.^[23]^

Bibliometrics allows exploration of the knowledge base, research hotspots, and frontiers of a particular field of study through quantitative and statistical analysis of the literature in that field.^[24–27]^ The technique presents the resulting knowledge structure through visualization, often being referred to as a “scientific knowledge map.”^[28]^ Compared to traditional literature reviews, bibliometrics can provide an objective and comprehensive overview for revealing emerging frontiers and providing recommendations for future research and decision-making.^[29]^ Traditional reviews relying on meta or subjective selection often result in limited research inclusion.^[30]^ Despite witnessing a steady increase in the number of publications on MG, to our knowledge, there have been no previous bibliometric analyses reported on this topic. Thus, we conducted the first bibliometric analysis of research on MG using the software programs VOSviewer 1.6.18, CiteSpace 6.1.R3, and the Online Bibliometric Analysis Platform (https://bibliometric.com/). Displaying vast amounts of literature data on a series of knowledge maps, our study offers clear insights on the knowledge base, hotspots, and frontiers of MG research.

## 2. Methods

### 2.1. Data source and retrieval strategy

**Figure 1.**
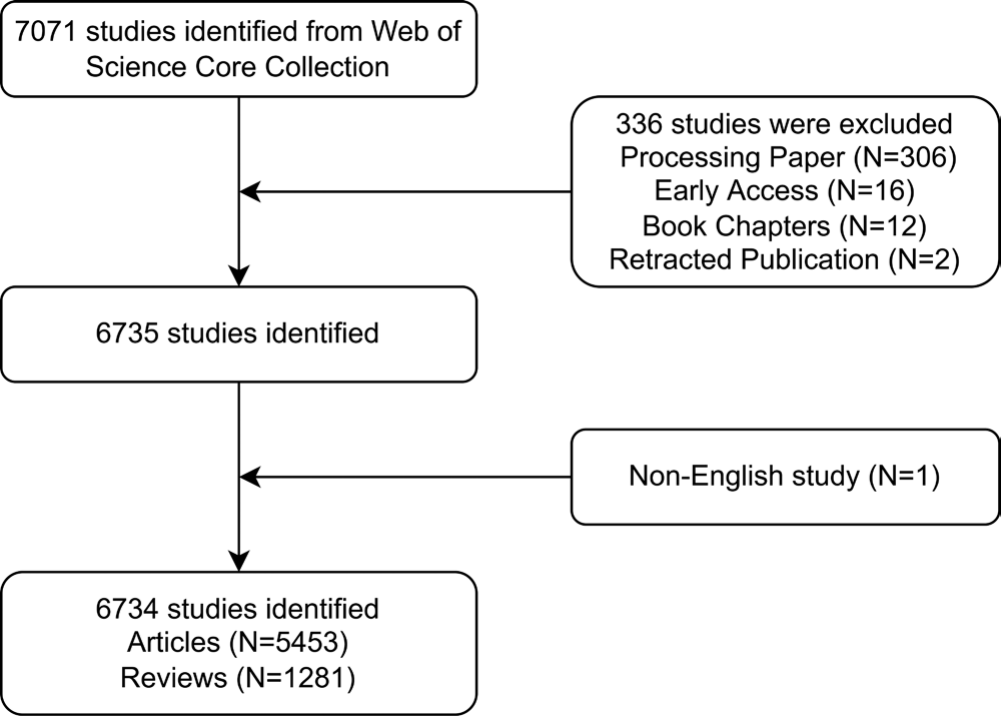
Flowchart of the literature screening process.

The literature records were retrieved and extracted on September 22, 2022, to ensure accurate inclusion and avoid any potential deviations resulting from updates to the Web of Science Core Collection (WoSCC) database. To enhance the accuracy of our retrieval, we combined subject headings with free words for the retrieval strategy, which followed the pattern: (((TS = (MG OR ocular MG OR generalized MG OR MuSK MG)) AND DT = (Article OR Review)) AND LA = (English)) AND DOP = (2002-01-01/2021-12-31). Of the initial 7071 documents retrieved, irrelevant studies, including processing papers (306), early access (16), book chapters (12), retracted publications (2), and those written in non-English languages (1), were excluded. Ultimately, 6734 papers were included, encompassing 5453 articles and 1281 reviews. The literature screening process is illustrated in Figure 1.

### 2.2. Bibliometric analysis

To conduct our analysis, all literature records were exported from the WoSCC database in plain text format and named “download_xxx.txt.” These files were then imported into CiteSpace 6.1.R3 software for the analysis of keywords clustering and burst, category co-occurrence and burst, author co-citation, references co-citation, timeline and burst, and journal dual-map overlay. The resulting data were imported into VOSviewer 1.6.18 and Scimago Graphica 1.0.24 software for further analysis, focusing on keywords co-occurrence, country/region, institution, author collaboration, country/region geographical distribution, and journal co-citation. Additionally, all literature records were exported in UTF-8 format and uploaded to the Online Bibliometric Analysis Platform for country/region collaboration analysis. The CiteSpace software was parameterized as follows: the time span was set from January 2002 to December 2021; Years Per Slice was set to 2; Node Types included keyword, category, cited author, and reference; Selection Criteria was set to top 50 each slice; Pruning included pathfinder, pruning sliced networks, and pruning the merged network with the remaining settings left as defaults. For the VOSviewer software, the following parameter settings were utilized: Normalization method was set to association strength; the minimum thresholds for the number of publications from countries/regions, institutions, and authors were set to 5, 40, and 30 respectively; the minimum threshold for the number of journal citations was set to 1200, while the minimum threshold for the frequency of keyword occurrence was set to 100.

## 3. Results and discussion

### 3.1. Annual publications and citations

**Figure 2.**
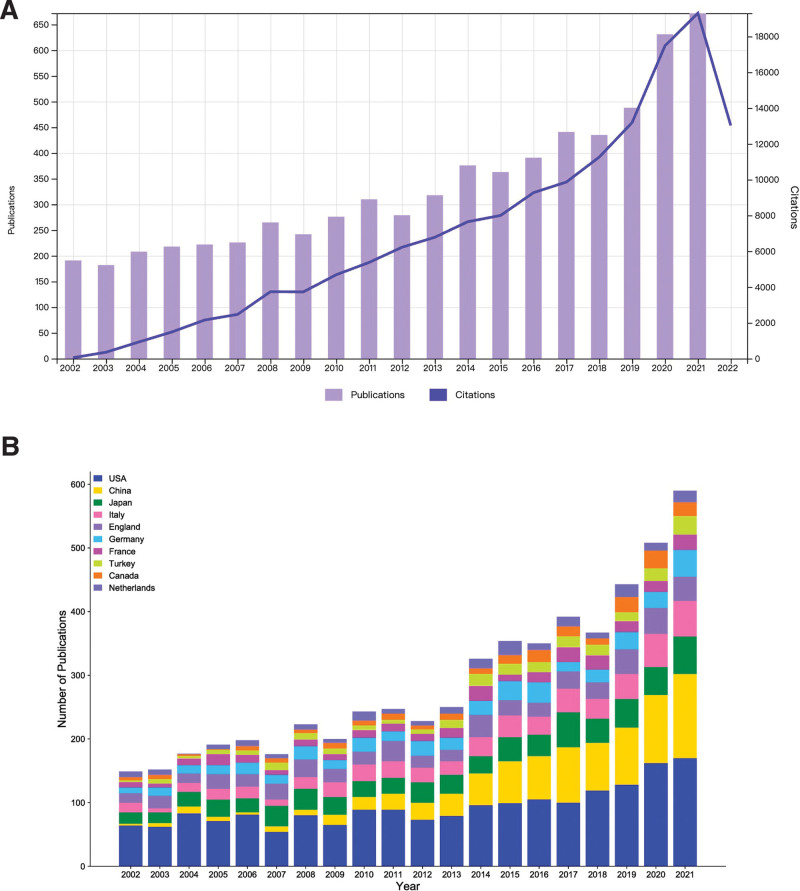
(A) The annual number of publications and citations on MG between 2002 and 2021. (B) The number of MG publications published in the top 10 countries/regions from 2002 to 2021. MG = myasthenia gravis.

This study included 6734 papers, comprising 5453 articles and 1281 reviews. Figure 2A displays the evolution of publications and citations over the past 2 decades, revealing an overall steady increase in annual publications and citations, indicating ongoing interest and investment in MG research. Notably, the past 2 years have seen annual publications reach over 600 and citation frequency exceeding 17,000, indicating a particularly enthusiastic and productive period in MG research. Figure 2B depicts the annual papers trend of the top 10 countries/regions contributing to the total number of publications, revealing the United States as a prominent contributor to the MG research field, consistently leading in both total and annual documents, with an upward trend in annual papers, albeit with some annual fluctuations.

China, as the second most productive country in MG research, has undergone 3 distinct periods of research. During the first period from 2002 to 2008, relatively few studies on MG were conducted, with an average of <10 papers published annually. The second period from 2009 to 2012 saw the development of MG research to a certain extent, with the average annual number of publications reaching over 20, and by 2012, China surpassed Italy, England, Germany, and France to rank third in terms of annual documents. The third period, from 2013 to 2021, has seen China research in MG advance rapidly, with the annual number of publications growing considerably, surpassing Japan to rank second in 2013 and exceeding 100 publications per year by 2020. China now ranks second after the United States in terms of both total and annual papers. It is noteworthy that since 2020, the combined annual publications of the United States and China have surpassed the combined annual publications of countries/regions ranked third to tenth in total publications, indicating that both countries have become leaders in MG research.

### 3.2. Analysis of nations/regions and organizations

These papers were published by 4708 organizations located in 107 countries or regions. The United States had the highest number of documents (1869, 27.76%), followed by China (847, 12.58%), and Japan (652, 9.68%) (Table 1). The United States ranked first in total citations, H-index, and total link strength, indicating that it is the leading contributor to MG research with significant academic influence and considerable cooperation with other countries or regions. Despite ranking tenth in total papers, the Netherlands ranked first in the average citations per paper, highlighting the high quality of Dutch research in the field of MG and its broad citation by many scholars.

**Table 1:** The top 10 countries/regions in the number of publications.

Rank	Countries/regions	Counts (%)	Total citations	Average citation per item	H-index	Total link strength
1	USA	1869 (27.76%)	58,518	31.31	105	912
2	China	847 (12.58%)	9628	11.37	40	225
3	Japan	652 (9.68%)	11,587	17.77	49	191
4	Italy	524 (7.78%)	16,480	31.45	65	546
5	England	508 (7.54%)	21,207	41.75	76	709
6	Germany	408 (6.06%)	17,882	43.83	69	546
7	France	281 (4.17%)	11,184	39.80	59	374
8	Turkey	241 (3.58%)	2980	12.37	27	144
9	Canada	221 (3.28%)	6459	29.23	40	269
10	Netherlands	213 (3.16%)	10,708	50.27	56	424

**Figure 3.**
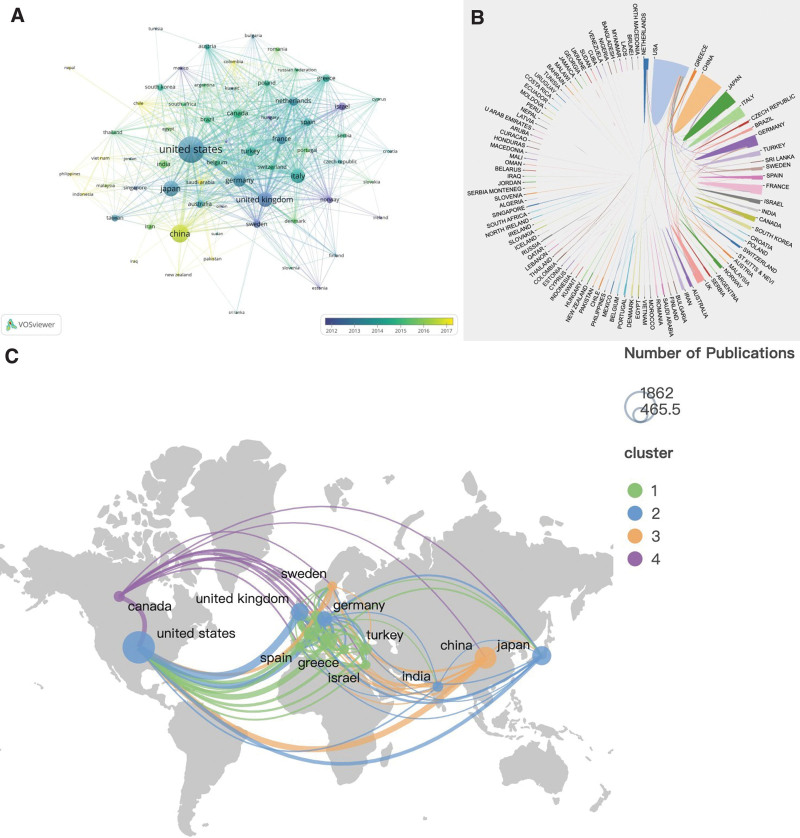
(A) The time overlay map of countries/regions cooperation network. (B) Knowledge map of countries/regions cooperation. (C) Geographical distribution of collaboration between countries/regions.

The time overlay map of countries/regions cooperation network (Fig. 3A) displays countries/regions with no <5 publications. The figure illustrates that research on MG began earlier in developed countries/regions such as the United States, Japan, Italy, England, and Germany, and a substantial amount of research was published in the early stages. On the other hand, China, as a developing country, started later but has significantly advanced in MG research, and an increasing number of papers have been published in recent years, making it the main researcher after the United States. Figure 3B provides a more intuitive view of the cooperation between countries/regions, indicating that the United States has established partnerships with multiple countries/regions, while China has limited cooperation with other countries/regions. Figure 3C clearly shows the intensity of cooperation between the main research countries/regions, revealing active partnerships between the United States and countries/regions such as England, Germany, China, Canada, and Japan. Although China ranks second in total publications, its total citations, average citations per study, H-index, and total link strength are all at a disadvantage, indicating lower research quality in MG, insufficient academic influence, and less cooperation. Thus, there is a need to strengthen international communication and cooperation to enhance research quality and academic influence, ensuring that the quality and quantity of research are equally important.

**Figure 4.**
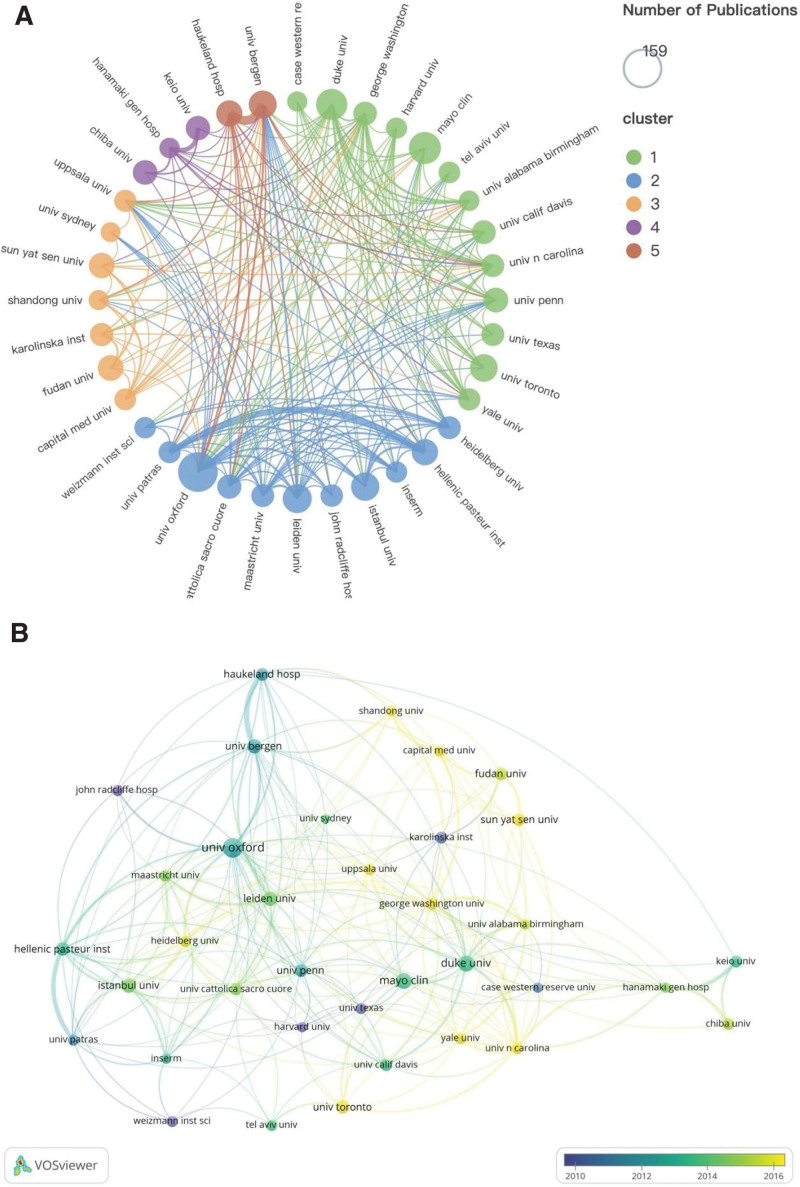
Knowledge map of institutions cooperation network. (A) The clustering map of institutional cooperation network. (B) The time overlay map of institutional collaboration network.

The time overlay map of the institutional collaboration network (Fig. 4B) displays institutions with at least 40 publications. The University of Oxford was the most productive institution (159, 2.36%), followed by Mayo Clinic (103, 1.53%), and Duke University (98, 1.46%) (Table 2). The University of Oxford in England also ranked the highest in H-index, reflecting its high academic standing in MG research. Despite ranking ninth in total number of documents, Haukeland Hospital in Norway ranked first in average citations per publication, indicating its high research level, quality publications, and widespread recognition and citation by researchers. The University of Bergen, also in Norway, ranked sixth in total publications but first in total link strength, confirming close collaboration with numerous research institutions. Fudan University, a renowned institution in China and the only Chinese institution ranked in the top 10 in terms of papers published in this field, started research on MG later and scored low in terms of average citations per publication, H-index, and total link strength, indicating insufficient research quality, academic influence, and cooperation with other institutions. Thus, strengthening cooperation and communication between institutions is necessary to enhance research quality and academic influence, along with improving research results’ quality in addition to increasing the number of papers.

**Table 2:** The top 10 institutions in the number of publications.

Rank	Institutions	Counts (%)	Average citation per item	H-index	Countries/regions	Total link strength
1	University of Oxford	159 (2.36%)	53.20	59	England	116
2	Mayo Clinic	103 (1.53%)	41.89	38	USA	36
3	Duke University	98 (1.46%)	60.95	40	USA	114
4	Leiden University	85 (1.26%)	58.86	40	Netherlands	68
5	Istanbul University	81 (1.20%)	19.92	22	Turkey	50
6	University of Bergen	79 (1.17%)	60.14	39	Norway	120
7	University of Toronto	77 (1.14%)	29.35	28	Canada	34
8	Hellenic Pasteur Institute	70 (1.04%)	32.76	24	Greece	112
9	Haukeland Hospital	69 (1.02%)	61.16	38	Norway	92
10	Fudan University	67 (0.99%)	13.18	18	China	40

The clustering map of the institutional cooperation network (Fig. 4A) displays institutions with no <40 publications. The figure reveals that the University of Bergen and Haukeland Hospital in Norway have the closest cooperation, and these 2 institutions have also established extensive collaboration with other institutions. Additionally, the cooperation between the University of Patras and Hellenic Pasteur Institute in Greece is the most frequent. Through the analysis of the cooperation between the 4 institutions in the 2 countries, it can be observed that inter-institutional international cooperation, especially between different countries, needs to be emphasized to promote the high-quality development of MG.

### 3.3. Analysis of journals

These included papers were published in 1612 journals. *Muscle & Nerve* was the most productive journal (321, 4.77%), followed by *Journal of Neuroimmunology* (232, 3.45%), and *Neuromuscular Disorders* (121, 1.80%) (Table 3). *Neurology* was the highest-rated journal in terms of average citations per study, H-index, and impact factor (IF), and was also the most frequently cited journal, indicating its high academic standing and quality in the field of MG research. The average H-index and IF of the 10 most productive journals were 123.7 and 5.664, respectively. Forty percent of these journals belonged to Q1, and 50% to Q2, demonstrating that most academic papers on MG are published in high-quality journals. Furthermore, these ten highly productive journals, all from western countries, have made significant contributions to the development of MG and have been widely recognized by scholars.

**Table 3:** The top 10 productive journals.

Rank	Journal	Counts (%)	Average citation per item	H-index	IF(2021)	Quartile in category
1	Muscle Nerve (United States)	321 (4.77%)	24.84	133	3.852	Q2
2	J Neuroimmunol (Netherlands)	232 (3.45%)	18.60	129	3.221	Q3
3	Neuromuscular Disord (United States)	121 (1.80%)	14.74	89	3.538	Q2
4	Neurology (United States)	107 (1.59%)	66.99	331	11.800	Q1
5	J Neurol Sci (Netherlands)	99 (1.47%)	17.98	124	4.553	Q2
6	Eur J Neurol (United States)	93 (1.38%)	33.99	112	6.288	Q1
7	J Neurol (Germany)	86 (1.28%)	24.63	122	6.682	Q1
8	Neurol Sci (Italy)	78 (1.16%)	11.22	64	3.830	Q2
9	Front Immunol (Switzerland)	75 (1.11%)	17.45	84	8.786	Q1
10	Front Neurol (Switzerland)	69 (1.03%)	7.91	49	4.086	Q2

IF = impact factor.

**Figure 5.**
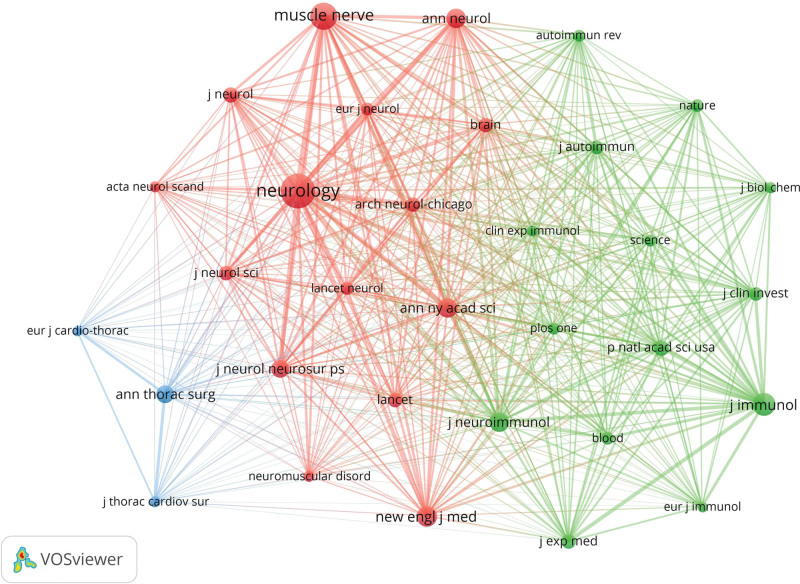
Knowledge map of co-cited journals network.

The journal co-citation network knowledge map (Fig. 5) displays journals that have been cited at least 1200 times. *Neurology* was the journal cited most frequently (n = 13,629), followed by *Muscle & Nerve* (n = 8437), and *Journal of Immunology* (n = 6233) (Table 4). *Neurology* also had the highest total link strength, indicating strong co-citation relationships with *Muscle & Nerve, Annals of the New York Academy of Sciences, New England Journal of Medicine, Journal of Immunology, Journal of Neuroimmunology, Annals of Neurology, Journal of Neurology Neurosurgery and Psychiatry*, among others. The average H-index and IF of the ten most cited journals were 344 and 24.969, respectively, with 70% in Q1 and 20% in Q2, indicating that high-quality journals are frequently cited. The *New England Journal of Medicine* had the highest H-index and IF, denoting its recognition as a premier high-quality journal in the academic community with high academic influence. American journals accounted for 80% of the top 10 cited journals, signifying their high academic influence and broad attention and citations. Consequently, priority should be given to reviewing literature from journals with high citation and productivity within this research field.

**Table 4:** The top 10 co-cited journals in terms of citation frequency.

Rank	Co-cited Journal	Citations	Total link strength	H-index	IF (2021)	Quartile in category
1	Neurology (United States)	13,629	482,554	331	11.800	Q1
2	Muscle Nerve (United States)	8437	266,683	133	3.852	Q2
3	J Immunol (United States)	6233	275,185	345	5.426	Q2
4	New Engl J Med (United States)	4812	173,264	933	176.079	Q1
5	Ann NY Acad Sci (United States)	4612	190,028	225	6.499	Q1
6	J Neuroimmunol (Netherlands)	4535	209,148	129	3.221	Q3
7	Ann Neurol (United States)	4515	218,003	273	11.274	Q1
8	J Neurol Neurosur PS (England)	3894	151,015	188	13.654	Q1
9	Ann Thorac Surg (United States)	3856	73,124	184	5.102	Q1
10	P Natl Acad Sci USA (United States)	2825	140,490	699	12.779	Q1

IF = impact factor.

### 3.4. Analysis of authors

**Figure 6.**
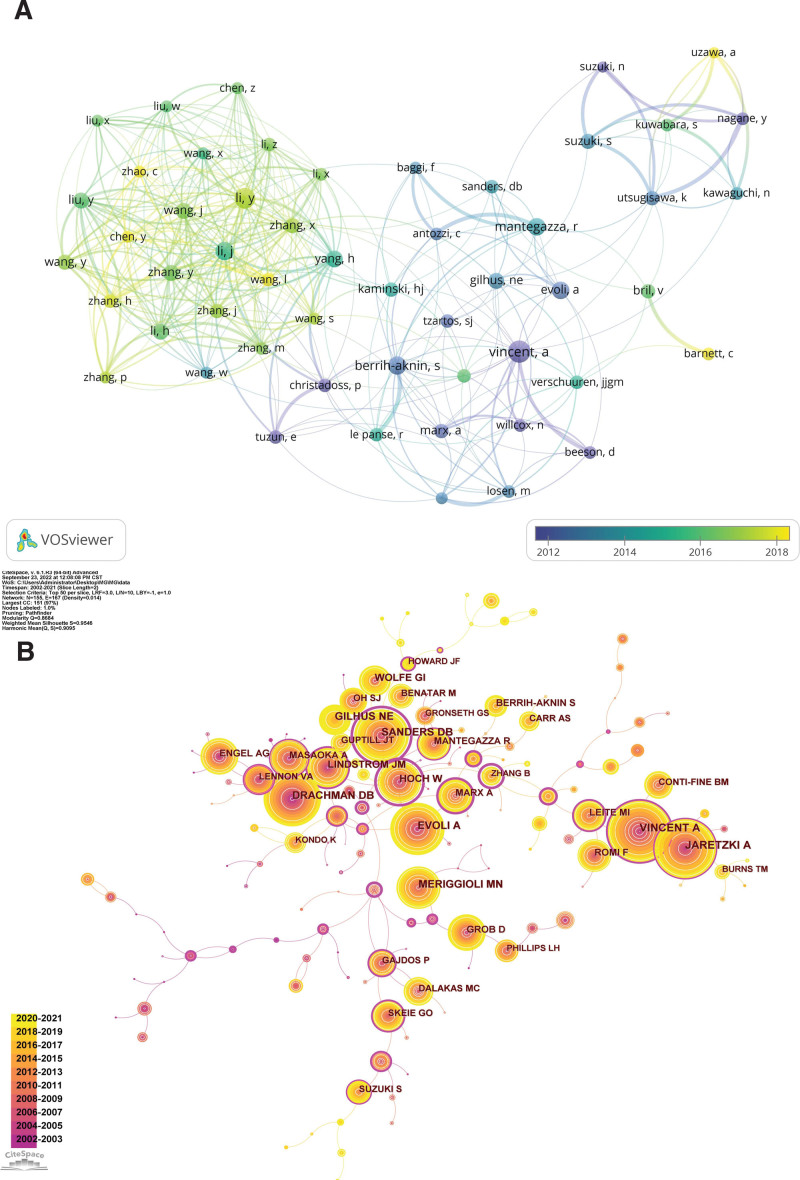
(A) The time overlay map of authors cooperation network. (B) knowledge map of co-cited authors network.

The 6734 publications were authored by 24,024 authors. The time overlay map of the authors collaboration network (Fig. 6A) presents authors with no fewer than 30 papers. Vincent A has produced the most papers (112, 1.66%), followed by Berrih-aknin S (78, 1.16%), and Evoli A (78, 1.16%) (Table 5). Among them, Vincent A from the University of Oxford in the UK was the most productive, cited, and had the highest H-index, indicating that he is a key contributor with a strong academic reputation in the field of MG research. One of his studies provided a comprehensive and systematic exposition of MG from epidemiology, clinical features, pathophysiology, diagnosis, and treatment, thereby laying a theoretical foundation for later research.^[31]^ Vincent A has active collaborations with Marx A, Gilhus NE, Berrih-aknin S, Evoli A, Mantegazza R, and Kaminski HJ. Sanders DB from Duke University ranked seventh in total publications but topped the list in average citations per paper. He was also a highly cited author, with high betweenness centrality, indicating his high academic level and that his high-quality publications have been widely recognized and cited by scholars, leading to significant academic influence in the MG research field. It is noteworthy that 90% of the top 10 authors in the publication are from Europe or North America, while China, the second-highest ranked country, has no top-ranked, highly productive authors.

**Table 5:** The top 10 authors in the number of publications.

Rank	Author	Counts (%)	Average citation per item	H-index	Countries/regions	Total link strength
1	Vincent A	112 (1.66%)	60.44	43	England	77
2	Berrih-aknin S	78 (1.16%)	44.79	34	France	77
3	Evoli A	78 (1.16%)	60.29	34	Italy	25
4	Mantegazza R	78 (1.16%)	34.65	29	Italy	86
5	Gilhus NE	73 (1.08%)	61.60	35	Norway	25
6	Kaminski HJ	60 (0.89%)	41.12	23	USA	20
7	Sanders DB	56 (0.83%)	71.29	35	USA	14
8	Bril V	54 (0.80%)	29.26	20	Canada	36
9	Marx A	54 (0.80%)	49.19	25	Germany	33
10	Suzuki S	53 (0.79%)	26.94	19	Japan	92

The author co-citation network knowledge map (Fig. 6B) displays authors whose citations are not <200 times. Vincent A was the most frequently cited author (1136), followed by Jaretzki A (1008), Sanders DB (908), Evoli A (828), and Drachman DB (793). Hoch W had the highest betweenness centrality (1.16), followed by Sanders DB (1.12), Tindall RSA (0.98), Palace J (0.96), and Higuchi O (0.58) (Table 6). Hoch W from the University of Houston was both the highly cited author and had the highest betweenness centrality, indicating that he plays a vital bridging role in MG research. Moreover, all 10 of the highly cited authors were from Western countries or regions, especially American authors, comprising 70% of the total. Sixty percent of the top 10 authors in the betweenness centrality ranking were from Europe, and 30% were from the United States. European and American authors, particularly the latter, are considered to have made significant contributions and have high academic prestige in the MG research field. Conversely, China MG research is relatively insufficient, with no core research groups or representative figures formed, indicating that Chinese researchers still have a long way to go in terms of adequate research. They should continuously enhance their research strengths, strengthen international exchanges and cooperation, and continue their in-depth research to publish high-quality research results.

**Table 6:** The top 10 co-cited authors in citation frequency and betweenness centrality.

Rank	Co-cited author	Citations	Location	Rank	Co-cited author	Centrality	Location
1	Vincent A	1136	England	1	Hoch W	1.16	USA
2	Jaretzki A	1008	USA	2	Sanders DB	1.12	USA
3	Sanders DB	908	USA	3	Tindall RSA	0.98	USA
4	Evoli A	828	Italy	4	Palace J	0.96	England
5	Drachman DB	793	USA	5	Higuchi O	0.58	Japan
6	Gilhus NE	723	Norway	6	Cavalcante P	0.55	Italy
7	Meriggioli MN	667	USA	7	Marx A	0.41	Germany
8	Hoch W	545	USA	8	Gajdos P	0.40	France
9	Lindstrom JM	492	USA	9	Hohlfeld R	0.39	Germany
10	Wolfe GI	486	USA	10	Strobel P	0.37	Germany

### 3.5. Analysis of cited references

**Figure 7.**
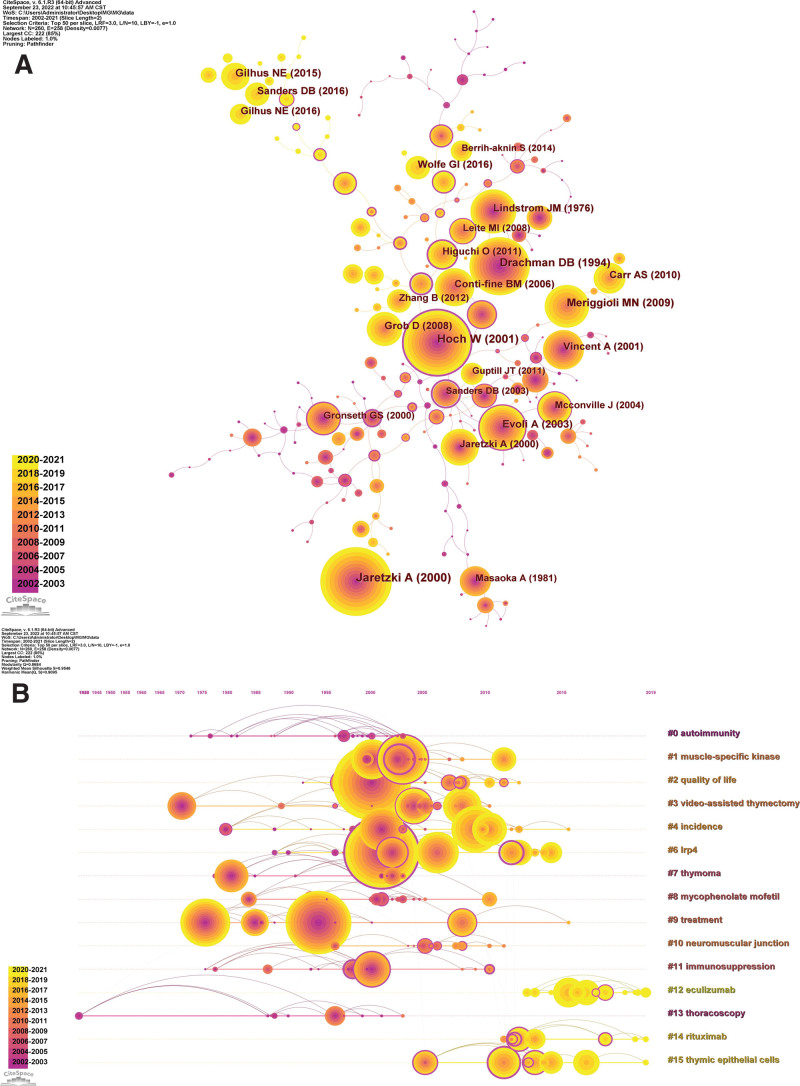
(A) Knowledge map of co-cited references network. (B) Timeline knowledge map of co-cited references.

The knowledge graph of the co-cited literature network (Fig. 7A) reveals papers that have been cited at least 150 times. The top 10 highly cited documents are presented in Table 7. The journals that published these 10 papers had an average IF and H-index of 45.715 and 382.9, respectively, with 90% in Q1 and 10% in Q2, indicating that these papers are high-level studies published in top-quality journals and are the fundamental knowledge base of the MG research field. The highest cited study was published in *Neurology* by Jaretzki A et al^[32]^ in 2000, proposing a classification system and definitions of treatment response. The second highest cited paper was published in *Nature Medicine* in 2001 by Hoch W et al,^[33]^ demonstrating that MuSK antibodies are involved in the pathogenesis of AChR antibody seronegative MG (SNMG). The third highest cited paper was published in 1994 by Drachman DB et al^[34]^ in the *New England Journal of Medicine*, systematically describing MG from clinical features, the NMJ, the acetylcholine receptor, immunopathogenesis, antibody-mediated mechanisms, role of lymphocytes, origin of the autoimmune response, diagnosis, and treatment. The *Lancet Neurology* published the fourth most cited literature by Meriggioli MN et al^[35]^ in 2009, outlining the epidemiology, immunopathogenesis, clinical presentation, diagnosis, and treatment of MG, including emerging treatment strategies. The fifth-ranked highly cited paper was published by Gilhus NE et al^[36]^ in the *Lancet Neurology* in 2015, providing an insightful review that systematically described the subgroup classification and treatment strategies for MG. The sixth most cited article was included in *Brain* by Evoli A et al^[37]^ in 2003, which described the clinical features associated with the presence of anti-MuSK antibodies in a series of patients with SNMG. The seventh highest cited paper was published in *Neurology* in 1976 by Lindstrom JM et al,^[38]^ analyzing the correlation between antibody titers and clinical parameters of MG and the value of antibody determinations as a diagnostic test for MG. The eighth-ranked study was a review published by Conti-Fine BM et al^[39]^ in 2006 in the *Journal of Clinical Investigation*, analyzing MG from a historical perspective, pathogenesis, diagnosis, therapeutic management, and future treatment strategies. The ninth ranked paper was an MG international formal consensus providing guidelines for clinicians, released by Sanders DB et al^[40]^ in *Neurology* in 2016. The tenth highest cited document was published in *Muscle & Nerve* by Grob D et al^[41]^ in 2008, analyzing the historical development of MG diagnosis and treatment and the effects of age, gender, thymectomy, and the presence of AChR antibodies on the clinical course of MG. The analysis of these 10 highly cited papers reveals that classification, pathogenesis, clinical features, diagnosis, and treatment are vital components of MG research.

**Table 7:** The top 10 co-cited references in citation frequency.

Rank	First author and publication yr	Citations	Journal IF (2021)	H-index	Quartile in category
1	Jaretzki A, 2000	681	Neurology (IF:11.800)	331	Q1
2	Hoch W, 2001	545	Nat Med (IF:87.241)	497	Q1
3	Drachman DB, 1994	489	New Engl J Med (IF:176.079)	933	Q1
4	Meriggioli MN, 2009	422	Lancet Neurol (IF:59.935)	259	Q1
5	Gilhus NE, 2015	343	Lancet Neurol (IF:59.935)	259	Q1
6	Evoli A, 2003	326	Brain (IF:15.255)	308	Q1
7	Lindstrom JM, 1976	297	Neurology (IF:11.800)	331	Q1
8	Conti-Fine BM, 2006	289	J Clin Invest (IF:19.456)	447	Q1
9	Sanders DB, 2016	278	Neurology (IF:11.800)	331	Q1
10	Grob D, 2008	268	Muscle Nerve (IF:3.852)	133	Q2

IF = impact factor.

Table 8 lists the top 10 references for betweenness centrality, which had an average IF and H-index of 19.448 and 253.2, respectively, and 90% of them were in Q1 and 10% in Q2, highlighting their high-quality and pivotal bridging role in the field. Through analysis, it can be observed that 3 studies demonstrated the role of LRP4 antibodies in the pathogenesis of AChR antibodies SNMG^[42–44]^ while 5 studies indicated that MuSK antibodies are involved in the pathogenesis of AChR antibodies SNMG.^[33,44–47]^ Two studies explored the efficacy of azathioprine and mycophenolate mofetil in the treatment of MG, respectively,^[48,49]^ while 1 publication analyzed the effects of thymus on the pathogenesis of different MG subtypes.^[50]^ Overall, the pathogenesis, therapy, MuSK antibodies, LRP4 antibodies, and thymus represent the research hotspots of MG to some extent. Furthermore, the timeline map of co-cited literature can visually exhibit the time span and transitions of the literature in each cluster by clustering the references, exploring the time evolution of research hotspots in this field.^[51]^ Figure 7B demonstrates that the references of MG research are separated into 15 clusters, with #12 eculizumab, #14 rituximab, and #15 thymic epithelial cells representing the current research hotspots.

**Table 8:** The top 10 co-cited references for betweenness centrality.

Rank	First author and publication yr	Centrality	Journal IF (2021)	H-index	Quartile in category
1	Pevzner A, 2012	0.97	J Neurol (IF:6.682)	122	Q1
2	Sanders DB, 2003	0.84	Neurology (IF:11.800)	331	Q1
3	Hoch W, 2001	0.73	Nat Med (IF:87.241)	497	Q1
4	Palace J, 1998	0.69	Neurology (IF:11.800)	331	Q1
5	Pasnoor M, 2010	0.59	Muscle Nerve (IF:3.852)	133	Q2
6	Higuchi O, 2011	0.46	Ann Neurol (IF:11.274)	273	Q1
7	Klooster R, 2012	0.46	Brain (IF:15.255)	308	Q1
8	Sanders DB, 2008	0.33	Neurology (IF:11.800)	331	Q1
9	Marx A, 2013	0.31	Autoimmun Rev (IF:17.390)	103	Q1
10	Verschuuren JJGM, 2013	0.31	Autoimmun Rev (IF:17.390)	103	Q1

IF = impact factor.

### 3.6. References with citation burst

**Figure 8.**
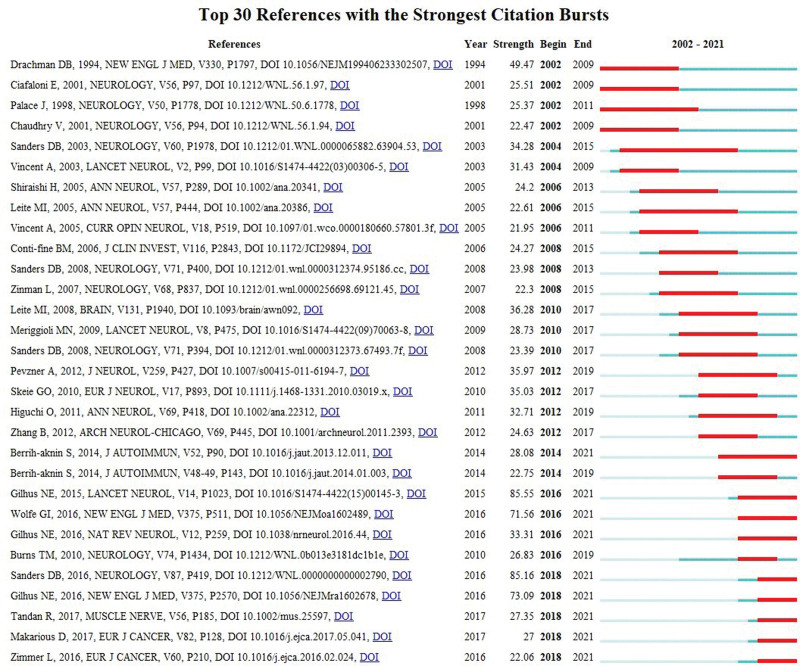
The top 30 references with the strongest citation bursts.

Citation burst refers to literature that is frequently cited within a short period, reflecting the research frontier and emerging trends.^[52]^ The top 30 references for burst strength were identified by adjusting the burst duration to 3 years (Fig. 8). Among them, 9 references with the burst time ending in 2021 are further discussed as they represent the trend of MG research. The reference with the highest burst strength was a review systematically describing subgroup classification and treatment strategies for MG, published by Gilhus NE et al^[36]^ in *Lancet Neurology* in 2015. The second-ranked burst study was published by Sanders DB et al^[40]^ in *Neurology* in 2016, an MG international formal consensus providing guidelines for clinicians. In 2016, The *New England Journal of Medicine* published the third-ranked paper by Gilhus NE,^[12]^ which analyzed MG from clinical and pathogenic variants, coexisting disorders, therapy, and future directions. The fourth-ranked study in terms of burst strength was published by Wolfe GI et al^[53]^ in the *New England Journal of Medicine* in 2016, confirming that thymectomy improved the 3-year clinical prognosis of patients with non-thymomatous MG. The publication ranked fifth in burst intensity was included in *Nature Reviews Neurology* by Gilhus NE et al^[54]^ in 2006, discussing the antibodies involved in the pathogenesis of MG. The sixth highest burst strength literature, published by Berrih-aknin S et al^[55]^ in the *Journal of Autoimmunity* in 2014, reviewed the latest concepts related to the pathophysiology of different subgroups of MG, as well as analyzed the mechanisms of action of immune, genetic, hormonal, and environmental factors on MG. The seventh highest burst strength study, published by Tandan R et al^[56]^ in *Muscle & Nerve* in 2017, showed that rituximab is a safe and effective treatment for MG, particularly for MuSK antibody-positive patients. The eighth highest burst strength research published in the *European Journal of Cancer* by Makarious D et al^[57]^ in 2017, stressed the importance of early identification and aggressive treatment of immune checkpoint inhibitor-related toxicities. Finally, the ninth study ranking in burst strength was published in the *European Journal of Cancer* by Zimmer L et al^[58]^ in 2016, which reported that anti-programmed cell death 1 antibodies could induce immune-related adverse events (irAEs). Analysis of these studies shows that understanding the pathogenesis, subtype classification, thymectomy, antibody assays, rituximab, toxicity of immune checkpoint inhibitors, and irAEs are major frontiers in MG research.

### 3.7. Analysis of subject categories

**Figure 9.**
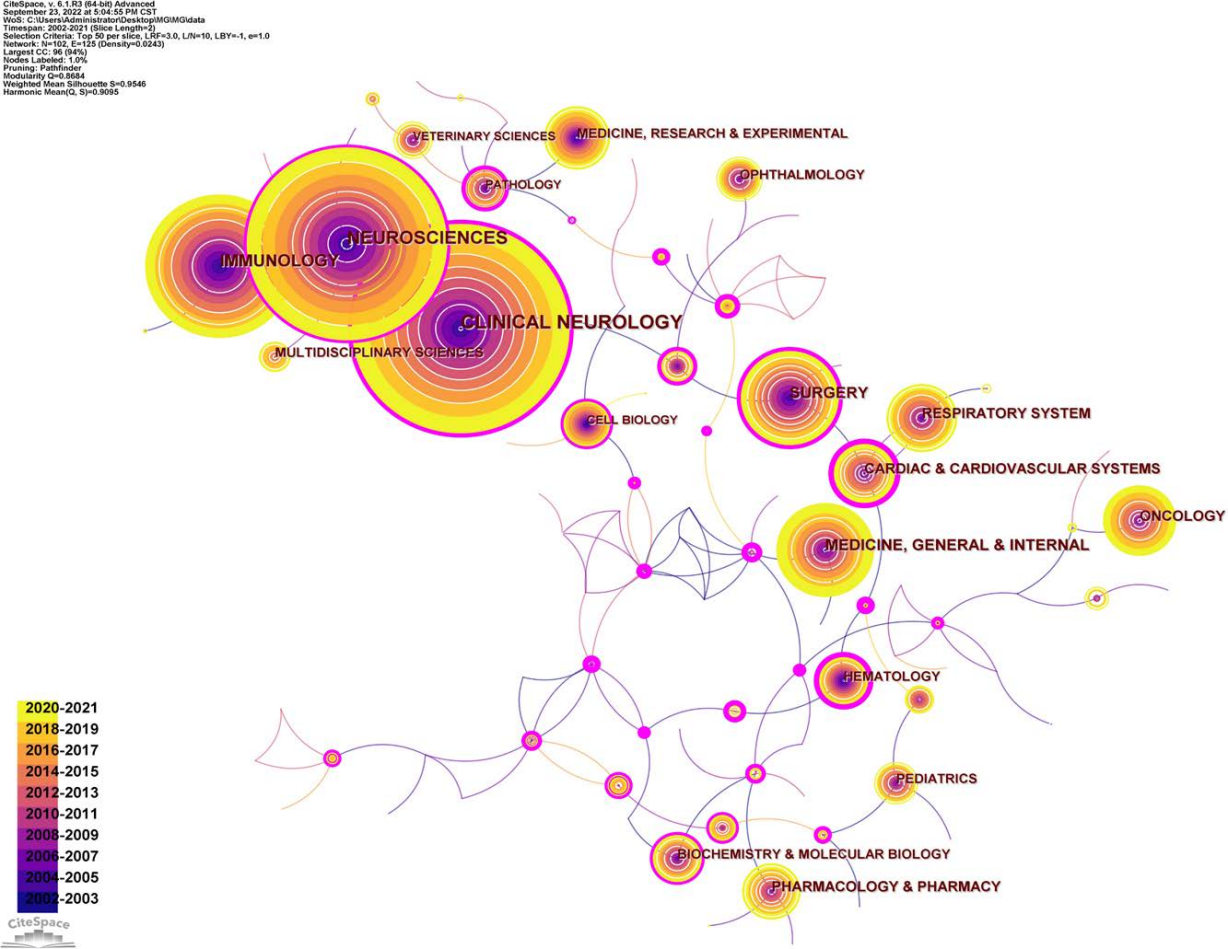
Knowledge map of category co-occurrence network.

**Figure 10.**
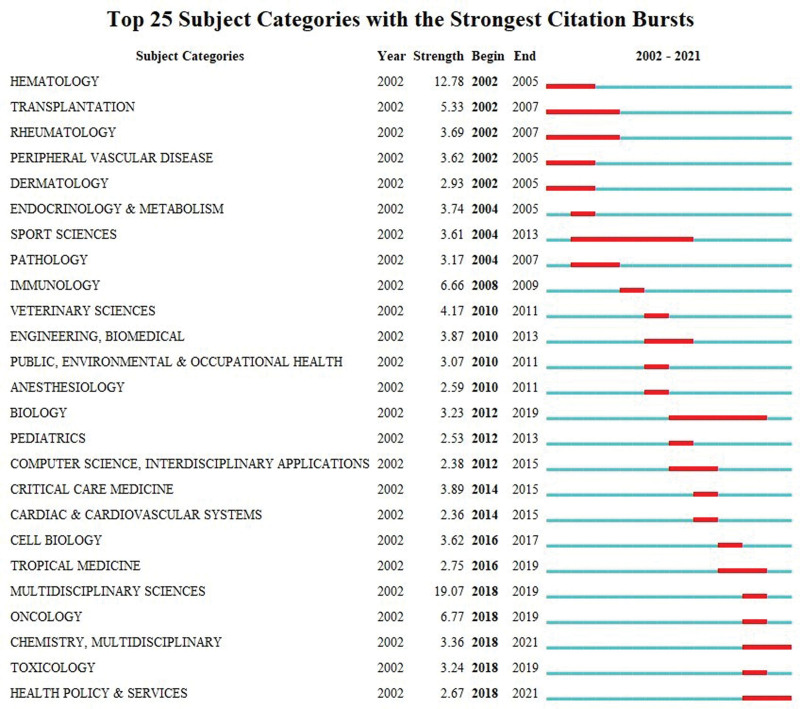
The top 25 subject categories with the strongest citation bursts.

The knowledge map of category co-occurrence network visualizes the subject categories that appear at least 100 times (Fig. 9). Clinical Neurology had the highest frequency of occurrence (2073), followed by Neurosciences (1732), Immunology (975), Medicine General Internal (564), and Surgery (521) (Table 9), indicating that these subject categories have extensively studied MG and are the main contributors to MG research. Health Care Sciences Services had the highest betweenness centrality (0.90), followed by Computer Science Interdisciplinary Applications (0.86), Engineering Biomedical (0.78), Transplantation (0.60), and Integrative Complementary Medicine (0.55) (Table 9), highlighting their crucial bridging roles in interdisciplinary research. The top 25 categories of burst intensity were identified by adjusting the burst duration to 1 year (Fig. 10), with burst time of 7 subject categories ending in 2019 or later. Therefore, Biology, Tropical Medicine, Multidisciplinary Sciences, Oncology, Chemistry Multidisciplinary, Toxicology, and Health Policy Services represent the frontier research disciplines in the field of MG.

**Table 9:** The top 10 subject categories in frequency and betweenness centrality.

Rank	Subject categories	Frequency	Rank	Subject categories	Centrality
1	Clinical Neurology	2073	1	Health Care Sciences Services	0.90
2	Neurosciences	1732	2	Computer Science Interdisciplinary Applications	0.86
3	Immunology	975	3	Engineering Biomedical	0.78
4	Medicine General Internal	564	4	Transplantation	0.60
5	Surgery	521	5	Integrative Complementary Medicine	0.55
6	Oncology	330	6	Peripheral Vascular Disease	0.54
7	Respiratory System	311	7	Urology Nephrology	0.53
8	Pharmacology Pharmacy	279	8	Hematology	0.51
9	Cardiac Cardiovascular Systems	253	9	Public Environmental Occupational Health	0.50
10	Medicine Research Experimental	238	10	Health Policy Services	0.50

### 3.8. Analysis of keywords

**Figure 11.**
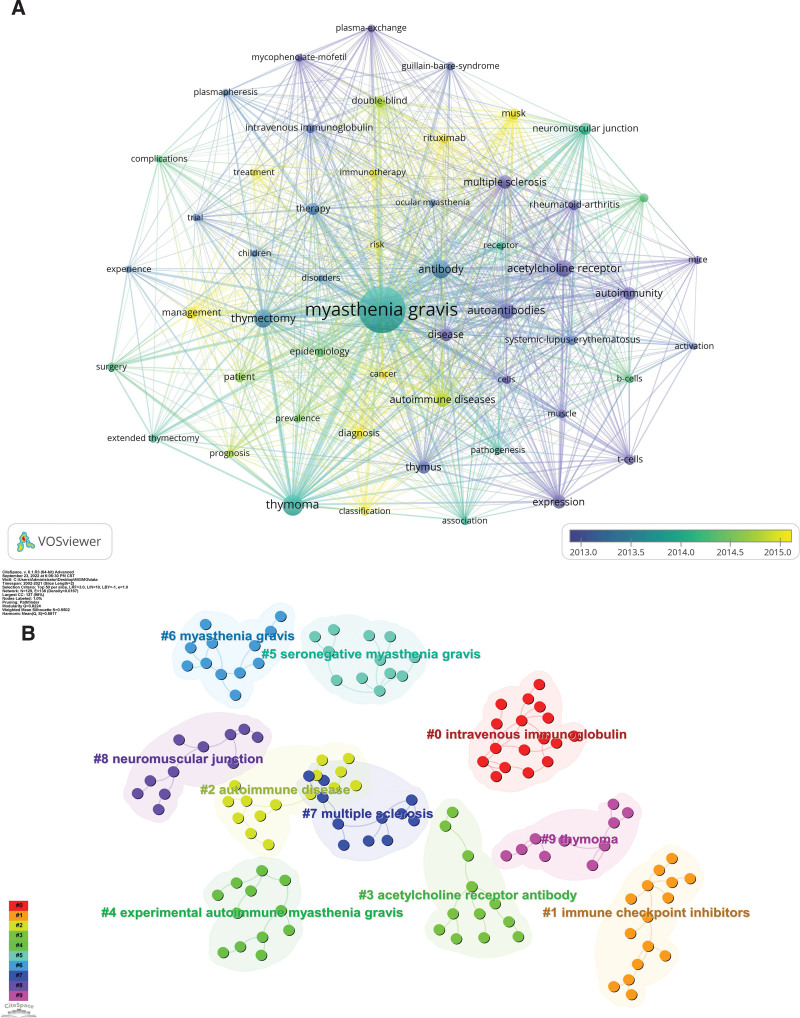
(A) The time overlay map of keyword co-occurrence network. (B) Knowledge map of keyword clustering network.

**Figure 12.**
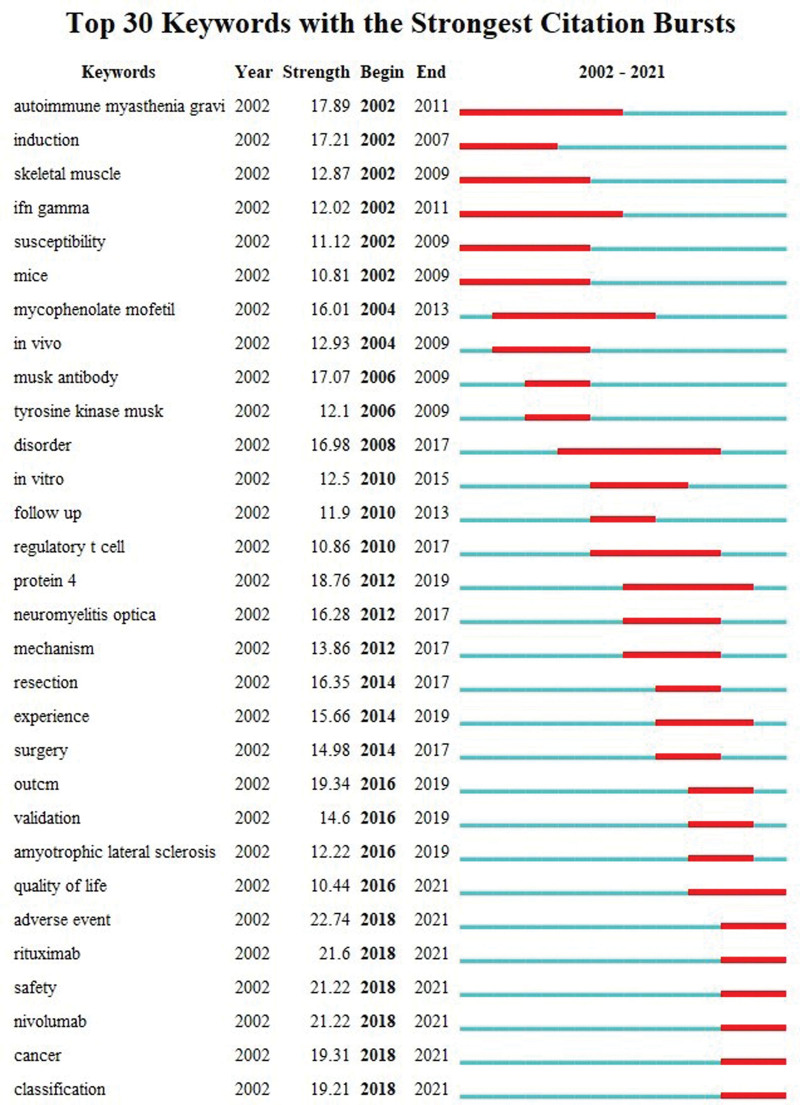
Top 30 keywords with the strongest citation bursts.

Keywords constitute the essence of the research topic and content of a paper, and analyzing their co-occurrence is helpful in exploring the research hotspots in the field.^[59–61]^ The keyword co-occurrence time overlay map illustrates keywords with a frequency of occurrence of no <100 times (Fig. 11A). It reveals that MG is the primary research topic, and the topics of MuSK, rituximab, immunology, treatment, risk, management, cancer, diagnosis, and classification have emerged recently, implying that they represent recent research hotspots. Figure 11B presents the keyword clustering analysis map indicating the 10 clustering labels determined by using the log likelihood radio algorithm. As a result, #0 intravenous immunoglobulin, #1 immune checkpoint inhibitors, #2 autoimmune disease, #3 acetylcholine receptor antibody, #4 experimental autoimmune MG, #5 SNMG, #6 MG, #7 multiple sclerosis, #8 NMJ, and #9 thymoma represent the primary research topics in MG. The top 40 high-frequency keywords are listed in Table 10, reflecting the main research content of MG.^[62]^ Moreover, the top 30 keywords with burst intensity were identified by adjusting the burst duration to 3 years (Fig. 12), with the burst time of 7 keywords ending in 2021, indicating that the quality of life, adverse events, rituximab, safety, nivolumab, cancer, and classification currently represent the latest MG research frontier. Through keyword co-occurrence, clustering, and burst analysis, the main research topics of MG were identified, and the research hotspots and frontier trends in recent years were explored.

**Table 10:** The top 40 keywords in frequency.

Rank	Keyword	Frequency	Total link strength	Rank	Keyword	Frequency	Total link strength
1	Myasthenia gravis	4480	10,351	21	Intravenous immunoglobulin	193	804
2	Thymoma	856	2469	22	Epidemiology	183	572
3	Thymectomy	685	2291	23	Rituximab	179	733
4	Antibody	655	2388	24	Guillain-barre-syndrome	173	453
5	Acetylcholine receptor	597	2173	25	Muscle	157	443
6	Autoantibodies	518	1937	26	Surgery	147	508
7	Autoimmune diseases	427	1484	27	Regulatory T-cells	146	487
8	Expression	377	1246	28	Plasma-exchange	145	594
9	Multiple sclerosis	376	1136	29	Receptor	144	497
10	Autoimmunity	334	1193	30	Pathogenesis	143	542
11	Thymus	319	1143	31	Association	143	516
12	Management	301	996	32	Classification	143	475
13	Neuromuscular junction	287	958	33	Prognosis	139	467
14	Therapy	277	1012	34	Mycophenolate-mofetil	137	685
15	Diagnosis	248	694	35	Cancer	137	445
16	Double-blind	243	1004	36	Extended thymectomy	134	466
17	Musk	238	995	37	Immunotherapy	132	464
18	T-cells	215	762	38	B-cells	127	502
19	Rheumatoid-arthritis	205	749	39	Mice	123	421
20	Systemic-lupus-erythematosus	202	658	40	Disorders	118	367

MuSK = muscle specific tyrosine kinase.

### 3.9. Analysis of treatment recommendations

Although MG is a NMJ disease caused by autoimmune factors, skeletal muscles are primarily affected. Therefore, in addition to interventions targeting the underlying causes of the disease, it is essential to focus on improving skeletal muscle function during treatment. As a type of motor tissue, skeletal muscle can be effectively improved through appropriate exercise, which is an intervention that contributes to preventing and treating MG. Regular exercise helps to increase muscle strength,^[63]^ enhance neurological function,^[64]^ promote better sleep quality,^[65]^ all of which are important factors in treating MG. Additionally, exercise can help maintain a healthy weight and reduce the risk of other health conditions that may aggravate MG symptoms. However, it is crucial to exercise with caution, and consult with a physician or physical therapist to develop a safe and effective exercise plan tailored to individual needs and limitations. Under appropriate guidance, exercise can become a valuable tool for treating MG.

## 4. Strengths and limitations

This study provides a comprehensive, systematic, and intuitive presentation of the knowledge framework and research status of MG research in the last 2 decades, using scientometric analysis. The study also explores the hotspots and frontiers of MG research through the analysis of highly cited literature, highly betweenness centrality literature, reference burst, and keyword co-occurrence, clustering, and burst. To our knowledge, this study represents the first bibliometric analysis of relevant literature in the field of MG. However, our research has some limitations. Firstly, we used data solely from the WoSCC database, which may have led to the exclusion of some relevant studies. Nevertheless, the WoS database is commonly employed for bibliometric studies.^[66–68]^ Secondly, we included only articles and reviews in English to ensure accurate software analysis. Finally, as new and relevant papers continue to emerge, recent high-quality research may be underestimated.^[69]^

## 5. Conclusions

Bibliometric research has revealed that the annual number of publications and citations in MG research has displayed an overall upward trend over the past 2 decades, with the annual number of publications and citations exceeding 600 and 17,000, respectively, since 2020. The United States was identified as the major contributor and most influential country in the field of MG research. The University of Oxford was the leading research institution with high academic standing. Vincent A from the University of Oxford was found to be the most published and cited author, with significant academic influence and outstanding contributions. *Muscle & Nerve* and *Neurology* respectively were the most published and cited journals. Clinical Neurology and Neurosciences were the main subject categories studied. The hot research topics in recent years include pathogenesis, eculizumab, thymic epithelial cells, immune checkpoint inhibitors, thymectomy, MuSK antibodies, risk, diagnosis, and management. The burst keywords quality of life, irAEs, rituximab, safety, nivolumab, cancer, and classification indicate the current research frontiers in MG.

## Acknowledgements

Thanks to all study participants for their cooperation.

## Author contributions

**Conceptualization:** Jiali Yang, Zhaomeng Hou.

**Data curation:** Jiaojiao Wu, Tingliang Han, Fangcun Li, Zhaomeng Hou.

**Formal analysis:** Jiali Yang.

**Funding acquisition:** Zhaomeng Hou.

**Investigation:** Jiaojiao Wu, Fangcun Li, Zhaomeng Hou.

**Methodology:** Jiali Yang, Jiaojiao Wu, Tingliang Han, Hua Lu, Shaoting Su.

**Resources:** Jiaojiao Wu, Zhaomeng Hou.

**Software:** Jiali Yang, Hua Lu, Leilei Li, Zhaomeng Hou.

**Supervision:** Tingliang Han, Leilei Li, Ping Jiang.

**Validation:** Hua Lu, Ping Jiang, Zhaomeng Hou.

**Visualization:** Zhaomeng Hou.

**Writing – original draft:** Jiali Yang, Zhaomeng Hou.

**Writing – review & editing:** Tingliang Han, Fangcun Li, Leilei Li, Shaoting Su, Ping Jiang.
